# Out of the blue: detection of a unique highly pathogenic avian influenza virus of subtype H7N5 in Germany

**DOI:** 10.1080/22221751.2024.2420723

**Published:** 2024-10-22

**Authors:** Ann Kathrin Ahrens, Anne Pohlmann, Christian Grund, Martin Beer, Timm C. Harder

**Affiliations:** Institute of Diagnostic Virology, Friedrich-Loeffler-Institut, Greifswald, Germany

**Keywords:** Avian influenza, laying hen, phenotype, mutation, low pathogenicity

The current situation of an avian panzootic caused by highly pathogenic avian influenza viruses (HPAIV) of the goose/Guangdong lineage, subtype H5N1, clade 2.3.4.4b, is attracting significant attention and resources from both operational animal disease control and infectious disease research forces [1]. Consequently, the scrutiny of the scientific community may, at least temporarily, shift away from other avian influenza viruses. The occurrence of low and high pathogenicity H7N9 AIV in China between 2013 and 2018 provides compelling evidence of the significant zoonotic potential of subtypes other than H5 [2]. We report here the isolated instance of an infection with a unique HP H7N5 AIV subtype in a chicken layer farm in Germany during the summer of 2024 also to highlight the continuing presence and potential importance of AIV subtypes other than H5.

Case report: In late June of 2024, a commercial poultry enterprise in Germany keeping about 90,000 chicken layers was affected by a sudden rise in mortality. Clinical signs in affected animals included reduced feed intake, listlessness and edematous head appendages prior to acute death within hours to 2–3 days after onset. Within two days mortality amounted to 6.000 hens (7%) before the remaining birds were culled and safely disposed of without delay [3]. Examination of combined nasal and cloacal swab samples of 80 clinically affected or dead birds gave evidence for the presence of an influenza A virus that was immediately subtyped as H7N5 with a polyclonal cleavage site (PEIPKRKKRGLF) by real time-RT–PCR and Sanger sequencing [4,5]. This cleavage site is identical to that of two older HP H7 isolates from outbreaks in laying hens in Germany and Australia (A/ck/Leipzig/137-8/1979 [GenBank L43913], A/ck/Victoria/224 /1992 [GenBank CY025078]) (https://www.offlu.org/wp-content/uploads/2022/01/Influenza-A-Cleavage-Sites-Final-04-01-2022.pdf). No further AIV subtypes have been detected by whole genome sequencing or by an RT-qPCR assay specific for 16 HA and 9 NA subtypes [4]. Virus isolation attempts readily yielded isolates from two samples with high virus loads in the first passage in embryonated chicken eggs. Clinical and virological investigations in the restriction zone did not give evidence for further spread of this virus or circulation of its presumed precursor. To date, sources and incursion routes of this outbreak remain enigmatic: no evidence for H7N5 viruses in wild birds or poultry has ever been obtained before in Germany and a report of the detection of an LP H7N5 virus in a mallard in neighbouring Netherlands (EPI_ISL_267186) dates back to 2014. Yet, wild birds seem to be the most likely source of introduction. It should be mentioned, that a similar outbreak, but featuring an unrelated HP H7N7, had occurred in 2015 in a layer farm in the same region; however, at that time, an LP precursor had been identified in a neighbouring layer farm [6].

Full genome sequences obtained from six samples from the HP H7N5 farm by an Oxford Nanopore Technology-based method (NGS) [7] were used to study phylogenetic relationship and identify putatively adaptive mutations of the viral genome segments. NGS-derived sequences showed further details in the cleavage site compared with the Sanger consensus sequence. In particular, position −4 of the cleavage site showed variation between K (aaa; 4 samples) and R (aga, 2 samples) (Figure 1). This might indicate that the virus in the farm is still in an adaptation phase following very recent (likely on the farm itself) spontaneous mutation from an LP precursor to a polybasic, HP pattern. Also, several distinct insertional mutation events might have occurred independently in that flock. Variable cleavage site motifs in different samples from HP H7 outbreak farms have already been described in the past [8]. Here, however, no evidence for an LP precursor site has been obtained from the NGS sequences, and, unfortunately, no serum samples were available for examination: the presence of H7 antibody-positive hens might have suggested that an LP precursor virus had been in circulation in the farm before the HP outbreak emerged.

**Figure 1.**
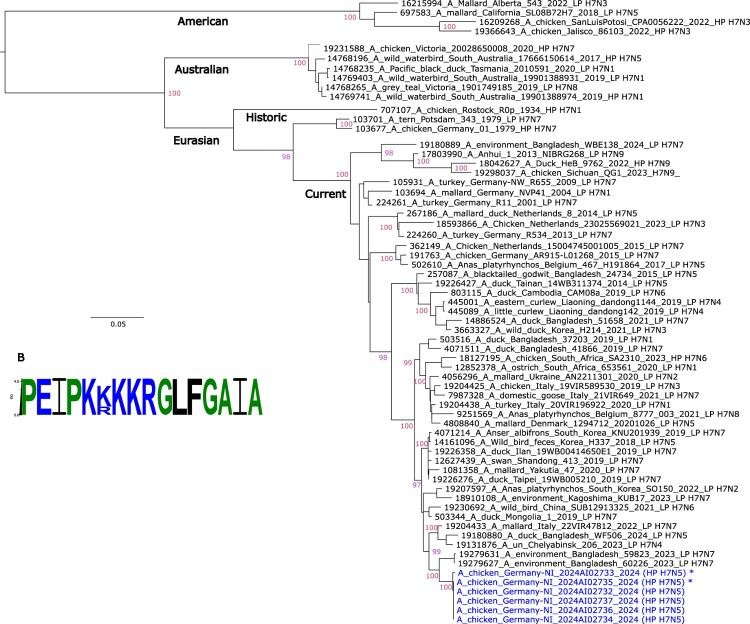
Phylogenetic relationship (A) of the H7 haemagglutinin nucleotide sequences of H7N5 HP viruses derived from clinical samples of an outbreak holding in Germany (blue colour; asterisks indicate successful virus isolation). (B) Sequence logo of the haemagglutinin cleavage site of six fully sequenced samples from the outbreak farm showing variable amino acid position −4 (K/R). Geographic and/or temporal restrictions are indicated at root-near branches. Notes: To investigate the phylogenetic relationship of the sequences the Rapid Barcoding kit (SQK-RBK114.24, Oxford Nanopore Technologies, Oxford, UK) was used in combination with the PromethION Flow Cell (FLO-PRO114M) on a PromethION 2 solo device with MinKNOW Software Core (v5.9.12). Live high-accuracy base calling of the raw data with Dorado (v7.3.11, Oxford Nanopore Technologies) was followed by demultiplexing, a quality check and a trimming step to remove low quality, primer and short (<20 bp) sequences. Therefore, predicaments associated with sequencing homopolymeric stretches such as the haemagglutinin cleavage site have been minimized. Multiple alignments for the HA segment were generated using MAFFT (v7.450). Maximum likelihood (ML) trees were estimated with IQ-Tree using a GTR-GAMMA model (GTR+F+G4) selected by ModelFinder. Bootstrap values obtained by the Ultrafast Bootstrap Approximation test are shown at nodes if >95. For further technical details and related literature see the supplemental file.

Sequences of all genome segments revealed similarities to avian influenza viruses from Asia and Europe (supplemental file). The HA H7 sequences from the outbreak farm formed a separate phylogenetic cluster characterized by a close relationship with very recent sequences from Bangladesh (e.g. A/environment/Bangladesh/59969/2023), Russia and Italy (Figure 1). In contrast, the LP H7N5 virus detected in 2014 in the Netherlands differed considerably with homology at the nucleotide level ranging between 91.0% (HA) and 98.8% (NS). The other segments showed similarities to Eurasian sequences from various wild bird species and different subtypes from the years 2020–2023 (supplemental file). A genome-wide search for putative adaptive mutations with a meaningful biological effect using FluMut [9] revealed in the viral protein polymerase basic 2 (PB2), part of the RNA-dependent RNA polymerase sub-complex, a substitution in position 526 from amino acid K to R. The K526R mutation in PB2 has been found to be enriched in human cases of H7N9 in China and of HP H5N1 in Indonesian patients, and in association with PB2 mutation E627K it enhanced viral replication in mammalian cells and was suspected to foster adaptation to humans [10]. This mutation was also detected in an outbreak of HPAI in cats in 2023 in Poland [11]. In addition, in the HA segment, all six samples show various substitutions including T143A affecting the receptor binding region of HA1 [12].

## Discussion

We report an isolated outbreak of HPAIV H7N5 in chicken layers that is unique globally and came “out of the blue” for the affected farm as well as for the related authorities. Failure to identify closely related, sympatric viruses of the same sub- and genotype in the databases re-emphasizes the underrepresentation of LPAIV in public databases. LPAIV of subtypes H5 and H7 may act as progenitors to HP phenotypes, and all subtypes may donate internal segments to HP viruses. Enrichment of sequence databases with LPAIV sequences requires enhanced surveillance, detection and sequencing activities which, in turn, must be based on intensified active monitoring programmes of wild birds. So far, surveillance efforts in the European Union have mainly focused on passive monitoring, which is inept to detect viruses such as LP H7N5, that presumably replicate in wild birds without severe clinical correlates. A similar example is the recently described novel subtype H19, whose few (*n* = 3) representatives also originate from active monitoring studies [13,14]. Nevertheless, this episode of a spontaneous, solitary HP H7N5 outbreak provides compelling evidence that, despite the current HP H5N1 panzootic, other AIVs with significant pathogenic potential continue to circulate and can emerge.

## Supplementary Material

Supplemental File.docx

## Data Availability

Sequence data are available in public INSDC under accession PP995008-PP995023 and PQ108614-PQ108645. Associated data are available in the public Zenodo repository under 10.5281/zenodo.13133875.
