# Supplementary material for: Out of the blue: detection of a unique highly pathogenic avian influenza virus of subtype H7N5 in Germany
Source: Emerg Microbes Infect. 2024 Oct 22;13(1):2420723. doi: 10.1080/22221751.2024.2420723 (PMC11552254; doi:10.1080/22221751.2024.2420723)
Supplement: Supplemental File.docx [file TEMI_A_2420723_SM1962.docx]

**Supplemental Table.** Closest related sequences for segments of HP H7N5 sample 2024AI02733.

| **Segment number** | **Segment** | **Percentage  homology** | **Closest relatives**  **(GISAID ID)** | **Pathotype^1^** |
| --- | --- | --- | --- | --- |
| 1 | PB2 | 98 | A/Anas_platyrhynchos/Belgium/827/2020 (A/H3N8), EPI_ISL_7591585 | n.a. |
| 2 | PB1 | 99 | A/greylag_goose/Austria/23145080-17/2023 (A/H6N1), EPI_ISL_18634821 | LP |
| 3 | PA | 98 | A/Anas_platyrhynchos/Belgium/827/2020 (A/H3N8), EPI_ISL_7591585 | n.a. |
| 4 | HA | 96 | A/environment/Bangladesh/59969/2023 (A/H7N7), EPI_ISL_19279628 | LP |
| 5 | NP | 98 | A/Mallard/Netherlands/3/2013  (A/H3N6), EPI_ISL_373088 | n.a. |
| 6 | NA | 98 | A/mallard duck/Netherlands/59/2015 (A/H6N5), EPI_ISL_328956 | LP |
| 7 | MP | 98 | A/mallard/Novosibirsk region/3445k/2020 (A/H1N1), EPI1849904 | LP |
| 8 | NS | 99 | A/barnacle goose/Sweden/SVA210511SZ0567/FB001840/M-2021 (A/H5N1), EPI1882787 | HP |

1 – Pathotype of the related sequence

n.a. – not applicable

**Supplemental Figure.** Phylogenetic trees of genome segments PB2 (A), PB1 (B), PA (C), NP (D), NA (E), MP (F) and NS (G) of two highly pathogenic H7N5 avian influenza viruses (blue color) representing an outbreak at a chicken layer farm in Germany, 2024.

Maximum likelihood estimations were carried out using the IQ Tree software (version 2.3.6, Linux; Nguyen et al., 2015). Appropriate models were selected using ModelFinder implemented in IQ Tree (Kalyaanamoorthy et al., 2017). Trees were drawn to scale by FigTree version 1.44 (https://github.com/rambaut/figtree /releases) and further refined using Inkscape 1.3 (https://inkscape.org/). Bootstrap values obtained by the Ultrafast Bootstrap Approximation test are shown at nodes if >95 (Minh et al., 2013).

**References**

Kalyaanamoorthy S, Minh BQ, Wong TKF, von Haeseler A, Jermiin LS. ModelFinder: fast model selection for accurate phylogenetic estimates. Nat Methods. 2017 Jun;14(6):587-589. doi: 10.1038/nmeth.4285

Minh BQ, Nguyen MA, von Haeseler A. Ultrafast approximation for phylogenetic bootstrap. Mol Biol Evol. 2013 May;30(5):1188-95. doi: 10.1093/molbev/mst024.

Nguyen LT, Schmidt HA, von Haeseler A, Minh BQ. IQ-TREE: a fast and effective stochastic algorithm for estimating maximum-likelihood phylogenies. Mol Biol Evol. 2015 Jan;32(1):268-74. doi: 10.1093/molbev/msu300.


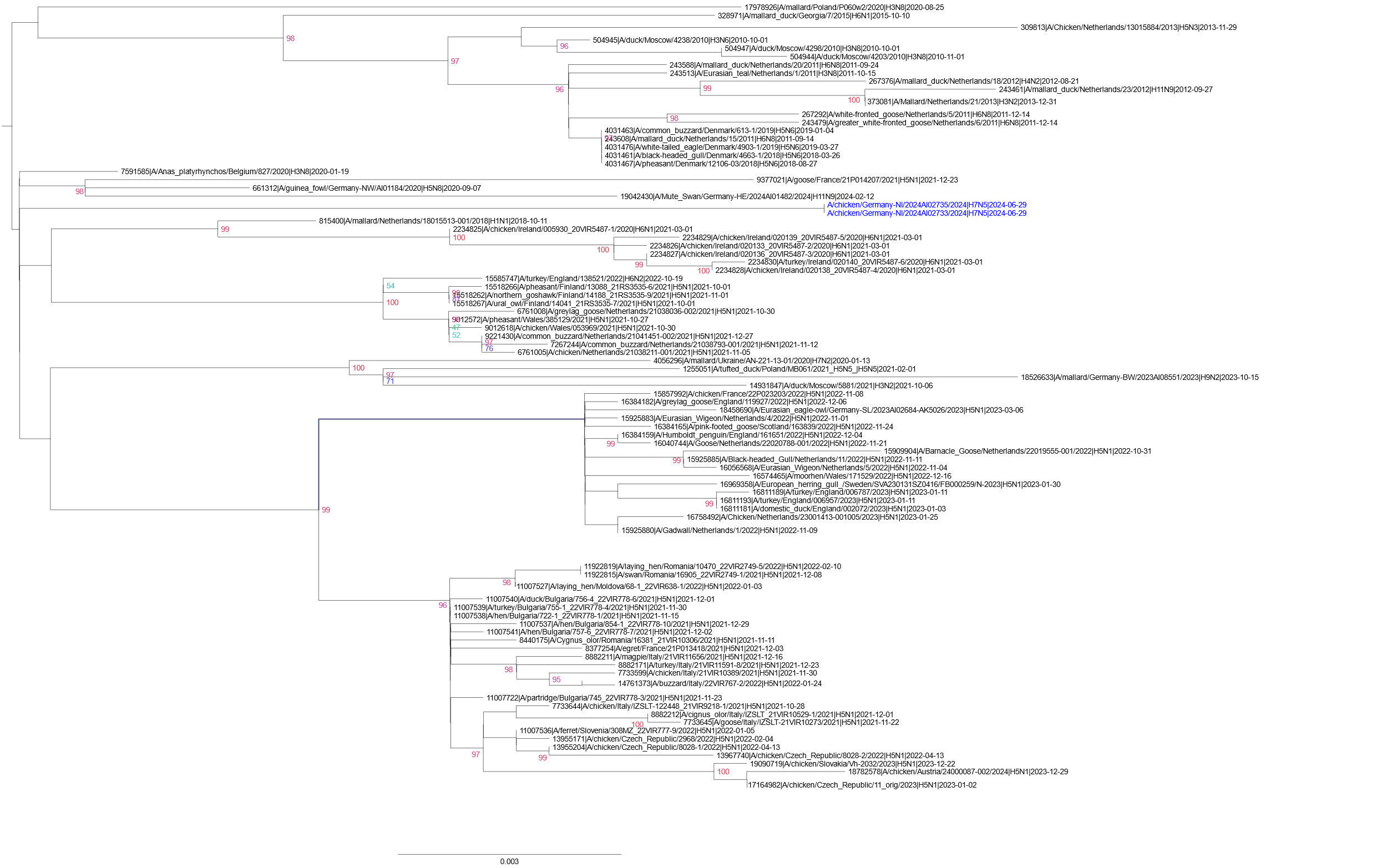


A.

A.


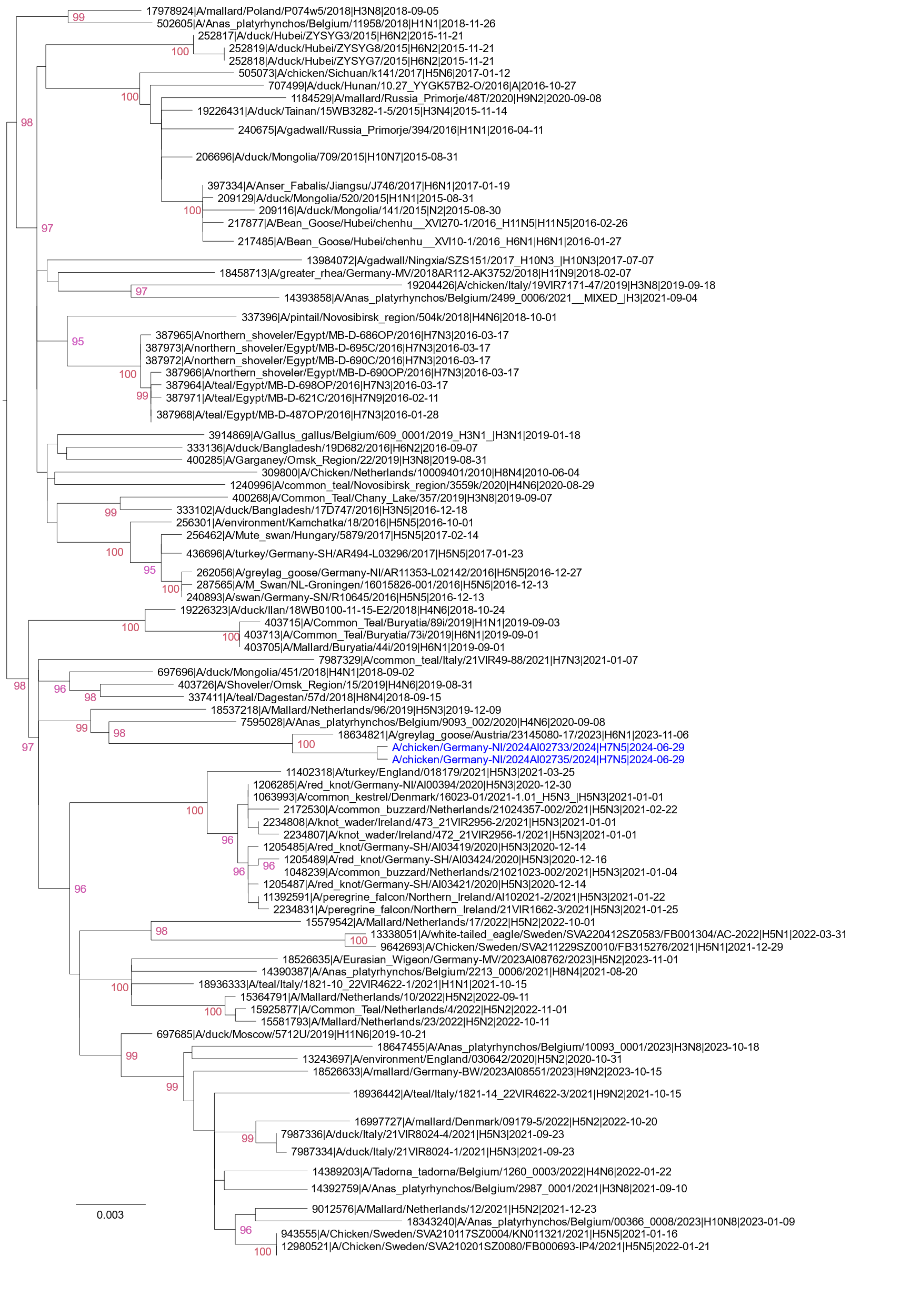


B.


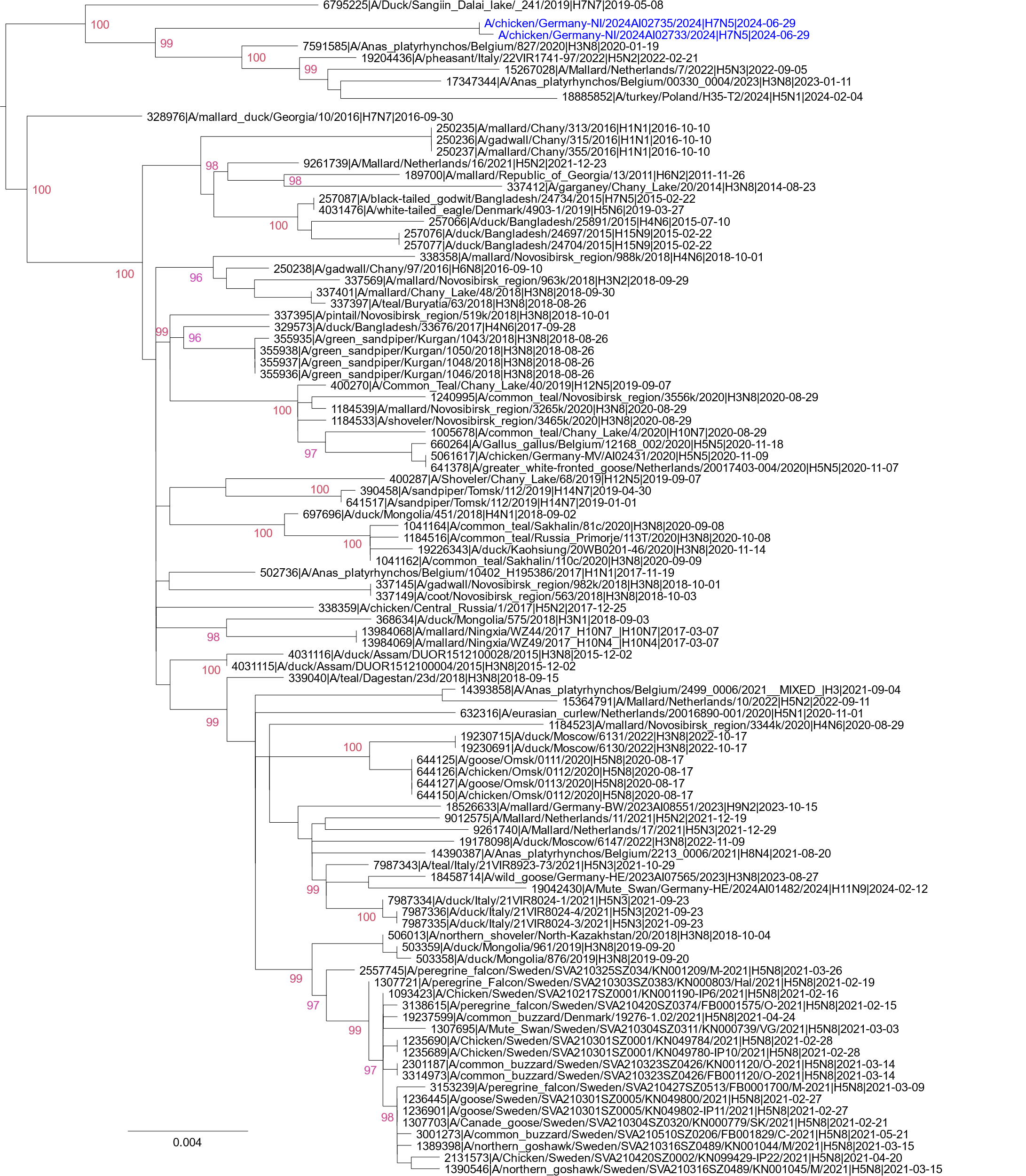


C.


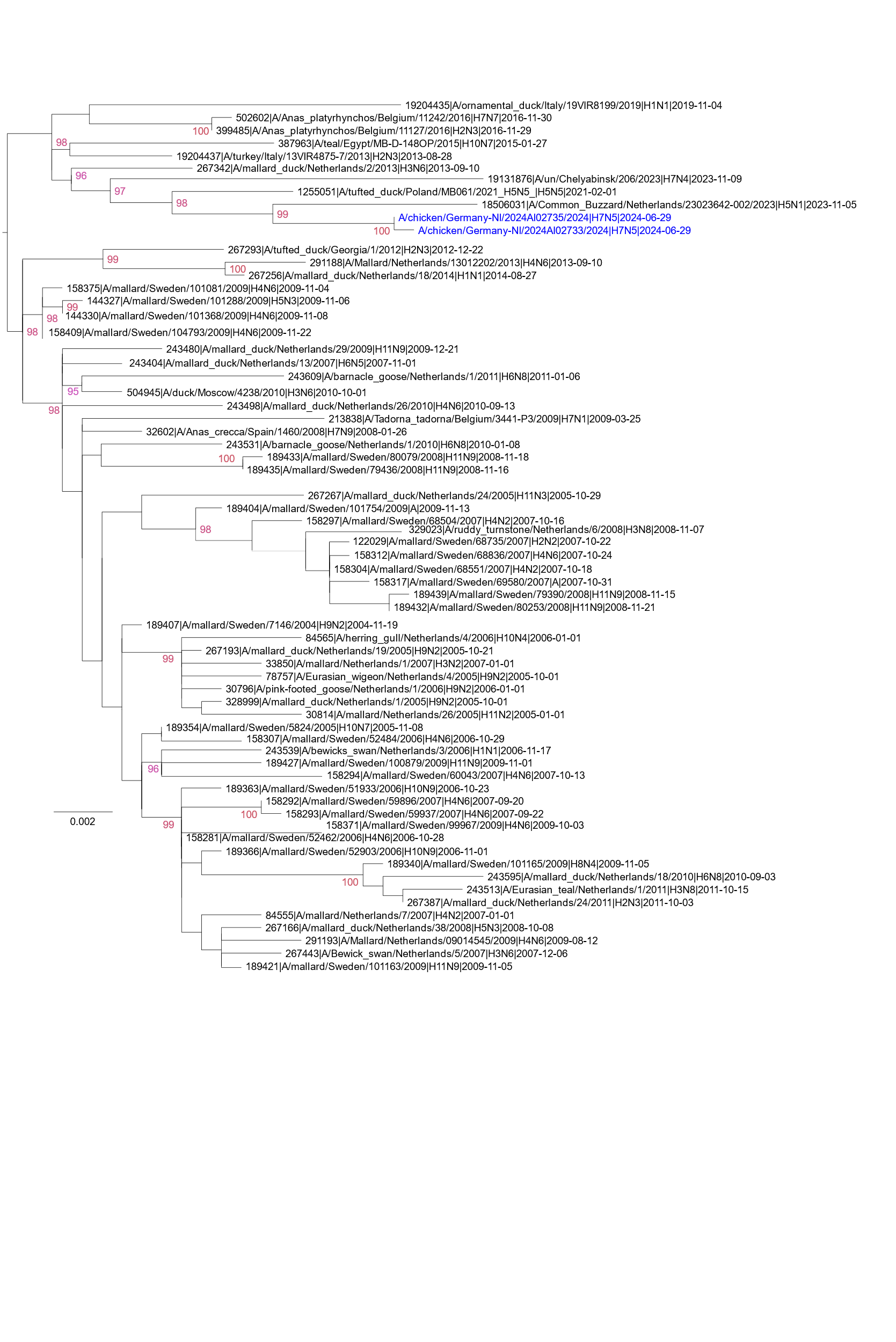


D.


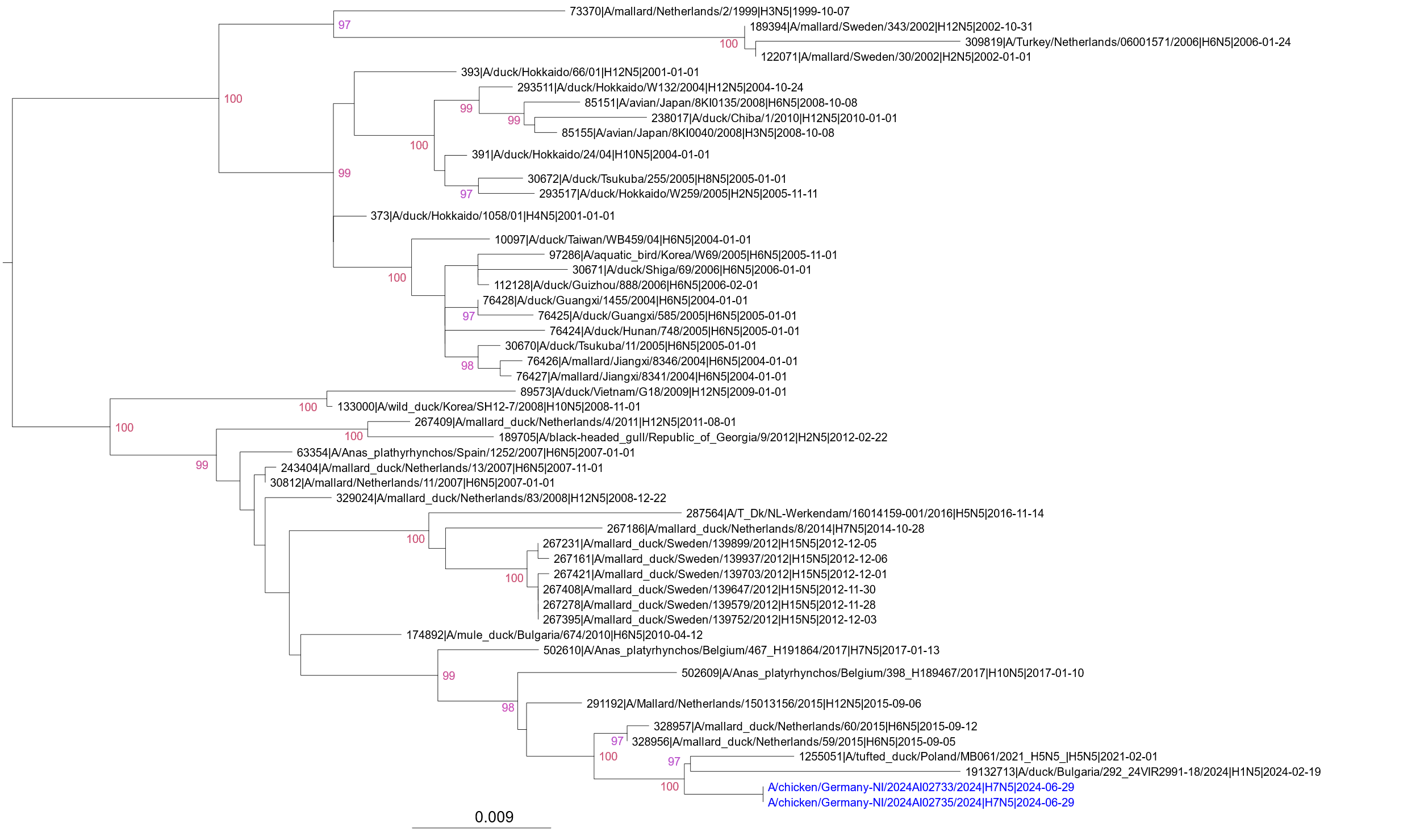


E.


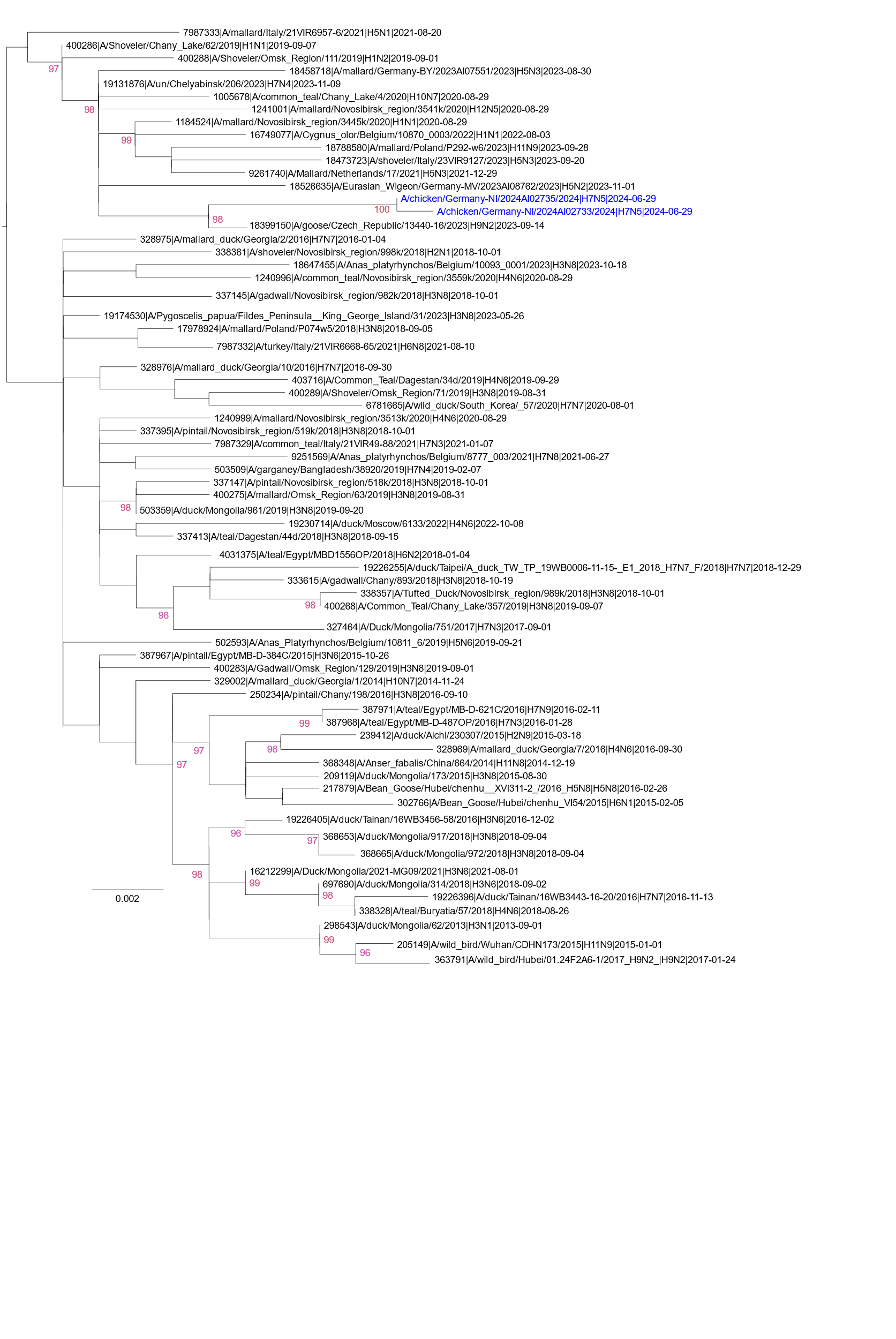


F.


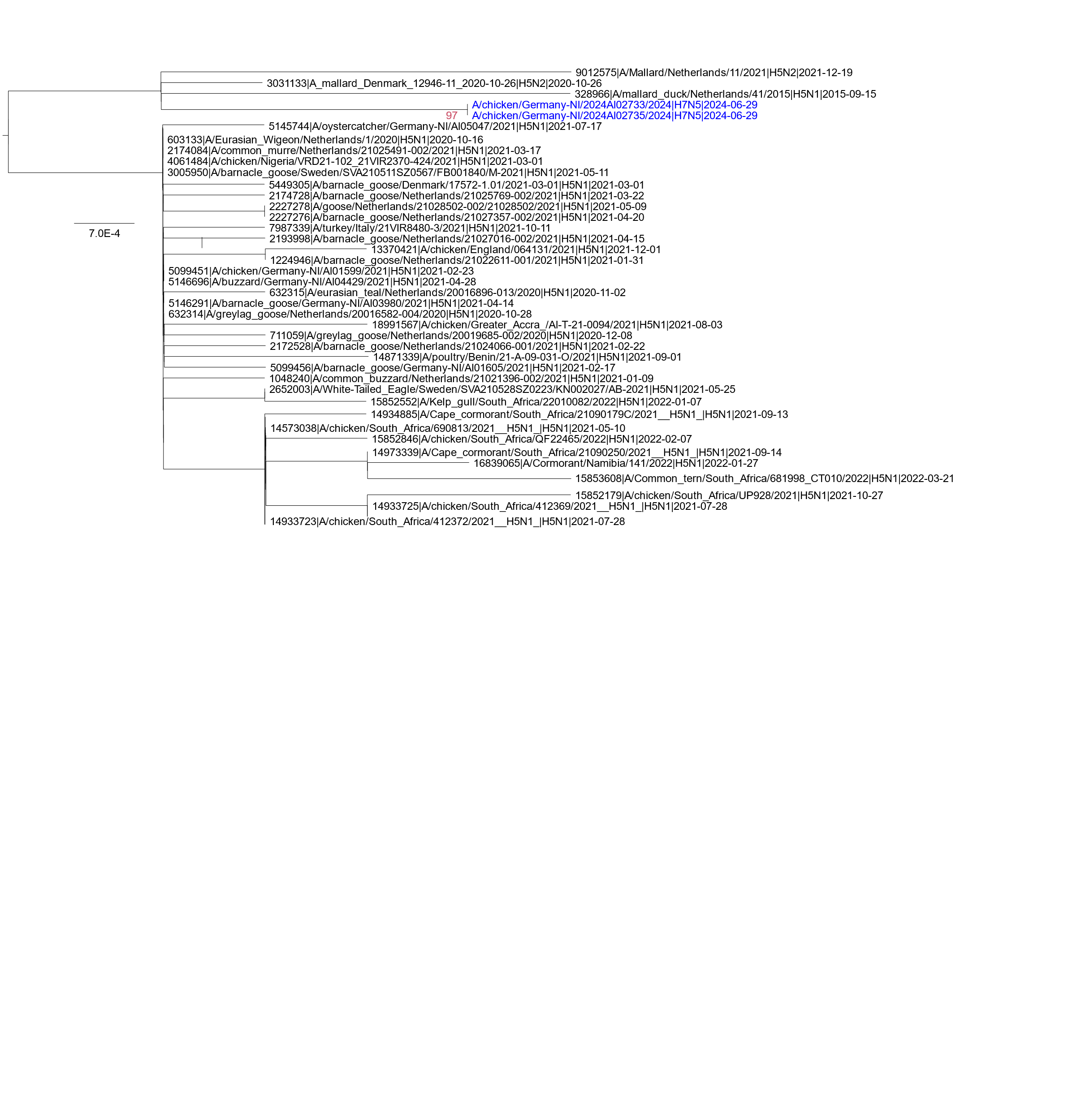


G.
